# The Visit of French Doctors to London

**Published:** 1905-01-07

**Authors:** 


### THE VISIT OF FRENCH DOCTORS TO LONDON.

THE visit of a number of French medical men to London in October last will be fresh in the recollection of readers of THE HOSPITAL, and it may be of interest to them to learn something of the impressions which the visitors carried away with them, as recounted in a series of articles which have appeared in the Paris *Bulletin Medical.* The whole of the articles are well worthy of perusal, and perhaps the following summary may induce some who read it to obtain the original papers. Each article deals with a separate aspect of the subject, so that it will be best to take them in order, noting the chief points in each as briefly as possible.

### I.—PRELIMINARY NOTE. By DR. JANICOT.

The party consisted of about 130, and more than 200 would have come if it had been known how well they would be received—the most eminent medical men in London, assisted by their wives, united to welcome them with open hearts, to show them everything, and to receive them into their families. They saw, he says, an organisation which contrasted sadly with that of the unfortunate hospitals of Paris. They were entertained at *déjeuners* and at a banquet, presided over by Sir William Broadbent, at which (and to this special attention is drawn) a long message from the King, addressed to the visitors in most flattering terms, was delivered. Dr. Janicot describes his scheme for recording the impressions of the visit, by entrusting each department to a different specialist, and regrets the impossibility, from want of the "sinews of war," of imitating in Paris the admirable arrangements of the majority of our institutions, while he thinks that we should view with envy the French maternity hospitals, and the "richesse cliniques incomparable" of St. Louis. He concludes by discussing the possibility of a return visit, and suggests that it should be arranged for next spring.

### II.—GENERAL IMPRESSIONS OF THE HOSPITALS. By DR. RENON.

Four main points are noted—the autonomy of our hospitals, the effects of private initiative, the abundant staff, and the cheerfulness (*gaieté*) of the wards. The first gives continuity and development (as seen in the marvellous out-patients' department of St. Thomas's); the second secures the constant support of the "grands hommes d'affaires," who give large sums annually while they live, and legacies when they die, and even persons of moderate means show their interest in the same way, on a smaller scale. Such a system would be impossible in France owing to the excessive centralisation of the administration. The abundant staff struck the visitors most; it amounts to one attendant to three patients, or even to two and a half, a position very different to the "notorious insufficiency" in France. We are held up to admiration as regards the education of the nurses, their status and their light, cheerful, healthy rooms, which would be the envy of many a physician in France. In the wards surprise is caused by the movable beds, and the wide spaces between them, their ample and decorative coverings, the cool colouring of the walls, the gay and coquettish tables covered with flowers and ornamental vases, all calculated to bring the sweets of home before the patients. The novel "cross" plan of University College Hospital is noted as giving the maximum of air and light; the comforts of the house-surgeons, the refrigeratin chambers, and the complete arrangements for laryngology ophthalmology, and radiotherapy are remarked upon. In a word, the hospital organisation of London is "bien superieure" to that of Paris. Dr. Renon concludes by regretting that the administration of the "Assistance Publique" did not send a representative to study the remarkable perfection of our hospital mechanism, especially as 45 millions (of francs) are about to be spent in rebuilding the hospitals of Paris.

### III.—OBSTETRIC HOSPITALS. By M. LEQUEUX.

M. Lequeux makes the most of the warm welcome he received, but he considers our maternity arrangements to be only worthy of a second-class town. The system at Queen Charlotte's is described in detail, with several comments, especially on the absence of separate labour-rooms for septic and aseptic cases. Regret is expressed at the profusion of hangings, the curtains to the cradles, the vases, flowers, and furniture, which please the eye, but contradict the most elementary principles of hygiene. Our system of attending mothers in their homes, instead of at the hospital, is mentioned, with its consequence, the absence of a special ambulance service. He criticises somewhat severely the training of midwives and maternity nurses in this country, remarking that the course of training is very short, and his article concludes with the discussion of several practical details and observations on the moral aspect of the facts referred to.

### IV.—SURGERY IN THE HOSPITALS. By DR. MANCLAIRE.

The writer of this article remained eight days in London, and was therefore able to see more than his colleagues. He describes each hospital visited in detail, and recounts the operations witnessed. At St. Thomas's he notes the complete use in the theatres of the aseptic system as a rarity among the places visited; also the absence of separation in the wards between septic and aseptic cases. The spacious wards are admired, and the rapid lifts are contrasted with the "litters borne by sleepy attendants" of Paris. The wards at University College are described as "petites, très propres, et tout à fait modernes." The London is described as a "new hospital," doubtless because the newer portions were visited. At the National Hospital for the Paralysed an operation in the new theatre is described as conducted with an extraordinary "sangfroid et methode," and the remarkable collection in the museum is referred to. Two other operations, at University College and the Hospital for Women, are described as "très methodiquement" and "avec beaucoup, de sangfroid et de méthode," the latter being one performed by Miss A. Blake. Several other hospitals are described, and in a general summary the writer describes the surgeons of London as adroit, quick, and operating with great sangfroid. The house-surgeons are referred to as very zealous and attentive, "in spite of their apparent phlegm." Special attention is drawn to the anæsthetists and their organisation, which does not seem to have any parallel in France. As regards the buildings and fittings the comments are nearly all favourable, especially on the absence of litters, but the wards are said to have rather too much furniture. Our out-patients' departments are noted as far larger and more complete than those in Paris, the hall of the Boucicaut Hospital being only equal to one of the small rooms of a London Hospital, and luxuries in Paris being considered as necessaries here. Our arrangements for operating directly the need is evident are warmly praised, as well as the completeness of the surgical and educational equipment in each hospital and their extremely practical nature. This is emphasised, with some humour, by the remark that, in France, one reads "Liberté, égalité, fraternité" over the door; in England, in the largest letters, "Supported by voluntary contributions"; and it is suggested that our title should be followed by "propreté, commodité, rapidité." The erection of our buildings in several stories, the use of lifts and telephones, the labels on the walls and doors showing the direction and position of each department, and the practical organisation generally, seem to greatly impress the writer in his concluding remarks.

V.—A MEDICAL VIEW OF THE HOSPITALS. By M. LOUPAULT.

This article refers, with favourable comments, to many points in our system mentioned before, including our voluntary subscriptions, the division into general and special hospitals, and general organisation. The construction of the wards, their ventilation, etc., are described in some detail. The arrangements for training and utilising the nursing staff are fully explained, and are contrasted with the inferior system of "infirmières" in France.

VI.—CHILDREN'S HOSPITALS. By M. APERT.

The writer comments on the frequent absence of any formal separation in London hospitals between children and adults, and notes that this is due in part to the existence of the medical schools attached to the general hospitals. That this system is satisfactory is due, he thinks, to the abundant and excellent nursing staff, but surprise is expressed at the absence of special wards for children in most cases. The absence of "asepsie" is remarked, but is explained by the perfect cleanliness of our hospitals, which renders it unnecessary. Our nurses' homes are specially admired, and again, for the third time, we have an encomium on our nurses, obviously recruited and educated quite differently from those of France. They have "activity, cleanliness, and elegant simplicity," and the impression given is that the English spend "enormous sums" on their production. (Quite true, M. Apert, but it is the nurses themselves who pay in most cases). The article concludes with some medical notes on children's diseases in England, and mentions the "enormous doses" given in certain cases, and describes our system of pasteurising milk, which seems to be less complete than that in France.

VII.—HOSPITALS FOR THE THROAT AND EAR. By DR. MALHERBE.

The writer says that the term hospital is hardly applicable to these institutions, which are more like private houses, with rooms in place of wards, containing two, four, or at most ten beds each. The rooms are often extremely pretty, the place is home-like, and, as in the large hospitals, everything is done to assure the comfort of the patient. The theatres are lit chiefly at the side, but as artificial light is a necessity in London, the want of a top light is not objectionable. The nurses and sisters are usually lodged on the upper floors. At the Throat Hospital, Golden Square, the system of operating by reflected light is noticed, a frontal mirror being used to reflect a fixed light instead of a frontal lamp. The extremely simple nature of the apparatus for sterilising instruments causes a little surprise. At the Throat and Ear Hospital the nature of the anæsthetics employed, and the care with which they are used, are noted with approval, and the article concludes with a short description of the new buildings of the Royal Ear Hospital for 30 beds, and with thanks to the doctors and surgeons who "did the honours" so agreeably.

(*To be continued.*)

THE Dumbarton Cottage Hospital was extended in the latter part of last year. Provision is now made for children for serious cases and private patients. An operating room has also been added.

